# Pulmonary Myeloid Cells in Mild Cases of COVID-19 Upregulate the Intracellular Fc Receptor *TRIM21* and Transcribe Proteasome-Associated Molecules

**DOI:** 10.3390/ijms26062769

**Published:** 2025-03-19

**Authors:** Andrea Henriques-Pons, Maria Clicia S. Castro, Vanessa S. Silva, Maiana O. C. Costa, Helena S. I. L. Silva, Maria Emilia M. T. Walter, Anna Cristina C. Carvalho, Alba C. M. A. Melo, Kary Ocaña, Marcelo T. dos Santos, Marisa F. Nicolas, Fabrício A. B. Silva

**Affiliations:** 1 Laboratory of Innovations in Therapies, Education and Bioproducts, Oswaldo Cruz Foundation, Rio de Janeiro 21040-900, Brazil; anna.carvalho@ioc.fiocruz.br; 2 Department of Informatics and Computer Science, State University of Rio de Janeiro, Rio de Janeiro 20550-900, Brazil; clicia@ime.uerj.br; 3 Scientific Computing Program, Oswaldo Cruz Foundation, Rio de Janeiro 21040-900, Brazil; vsantos223@gmail.com; 4 Computational Modeling Department, National Laboratory for Scientific Computing, Petropolis 15651-075, Brazil; maiolivei@gmail.com (M.O.C.C.); karyann@lncc.br (K.O.); msantos@lncc.br (M.T.d.S.); marisa@lncc.br (M.F.N.); 5 Department of Computer Science, University of Brasilia, Campus Universitário Darcy Ribeiro, Brasilia 70910-900, Brazil; helena.sils@gmail.com (H.S.I.L.S.); mariaemilia@unb.br (M.E.M.T.W.); alves@unb.br (A.C.M.A.M.)

**Keywords:** Fc receptors, COVID-19, scRNA-seq, inflammatory response, TRIM21

## Abstract

Much remains to be understood about COVID-19, but the protective role of antibodies (Igs) is widely accepted in SARS-CoV-2 infection. Igs’ functions are mainly carried out by receptors that bind to their Fc portion (FcR), and less attention has been dedicated to the cytoplasmic members of this family. In this work, we used single-cell RNA sequencing (scRNA-seq) data to discern cell populations in bronchoalveolar lavage fluid obtained from healthy individuals and patients with mild or severe COVID-19. Then, we evaluated the transcription of neonatal FcR (FcRn, *FCGRT* gene) and tripartite motif-containing protein 21 (*TRIM21*) and its downstream signaling components. The TRIM21 pathway is vital for virus infections as it has a dual function, leading opsonized viruses to degradation by proteasomes and the activation of innate inflammatory anti-virus response. The transcriptional level of *FCGRT* showed no statistical differences in any cell population comparing the three groups of patients. On the other hand, *TRIM21* transcription was significantly higher in myeloid cells collected from patients with mild COVID-19. When comparing mild with severe cases, there was no statistical difference in *TRIM21* transcription in lung adaptive lymphoid cells and innate lymphoid cells (ILC). Yet, we analyzed the transcription of all downstream signaling molecules in myeloid and, as most cells expressed the receptor, in adaptive lymphoid cells. Moreover, ILCs from mild cases and all cell populations from severe cases were missing most downstream components of the pathway. We observed that members of the ubiquitin–proteasome system (UPS) and other components associated with TRIM21 proteasomal degradation were transcribed in mild cases. Despite the transcription of the danger sensors *DDX58* and *IFIH1*, the transcriptional level of inflammatory *IL1B* and *IL18* was generally very low, along with the *NLRP3* danger sensor, members of the NF-κB pathway, and *TNF*. Therefore, our data suggest that TRIM21 may contribute to SARS-CoV-2 protection by reducing the viral load, while the inflammatory branch of the pathway would be silenced, leading to no pathogenic cytokine production.

## 1. Introduction

The COVID-19 pandemic has deeply affected the world and reaffirmed the importance of front-line scientific research [1]. Much remains to be understood about the immune response triggered by SARS-CoV-2, including the variation in immunological memory for long-term protection, why some patients develop more aggressive forms of the disease, and the cellular and molecular mechanisms underlying disease control [2]. However, it is common knowledge that the immune response is critical for restraining infection and disease spread. In this scenario, antibodies are pivotal in the protective cellular and molecular pathways triggered by natural infection, vaccine-based and other immunotherapeutic strategies used to combat COVID-19.

Antibodies perform multiple functions, and their Fc portion carries out most of them [3]. Immunoglobulins (Igs) bind specifically to pathogen epitopes via their hypervariable Fab portion and can prevent them from entering or damaging cells through neutralization. Moreover, the Complement cascade can be activated after Ig binding, leading to the lysis of pathogens or infected cells. Igs can also trigger the phagocytosis of opsonized pathogens and antibody-dependent cell cytotoxicity (ADCC), which are mediated by their Fc portion [4]. All Fc-mediated Ig functions are carried out by Fc receptors (FcR); among them, the FcRs for IgG (FcγR) are the most relevant. The FcγR family comprises activating the inhibitory receptors bearing immunoreceptor tyrosine-based activation motif (ITAM) or immunoreceptor tyrosine-based inhibition motif (ITIM), respectively. Multiple cell types express them and have well-described signaling pathways [5]. There is FcαR for IgA, FcμR for IgM, and other membrane-bound FcRs [6], but cytoplasmic FcRs are less explored.

Two FcRs are found in the intracellular environment: the neonatal FcR (FcRn, *FCGRT* gene) and the tripartite motif-containing protein 21 (TRIM21). Despite its name, FcRn is widely expressed throughout human adult life. It cycles between the plasma membrane and within intracellular endosomes, binding to IgGs under acidic but not neutral pH conditions. When the extracellular environment is neutral, IgG must be endocytosed and coupled to FcRn in acidic endosomes. Then, FcRn—IgG complexes can be sorted into the plasma membrane for recycling, while other cargo components are targeted for lysosomal degradation. The IgGs are released once reexposed to a neutral extracellular medium [7]. This process contributes to the unusually long half-life of circulating IgG and is likely one of the bases for many IgG-based infusion treatments [8].

The other intracellular FcR participates in a more complex cellular response, leading to pathogen destruction in proteasomes and inflammation. TRIM21 is an E3 ubiquitin ligase, a homodimer that forms a 1:1 complex with an antibody [9], and at least 24 TRIM21 homodimers must bind to an adenovirus particle for full virus neutralization [10]. The degradation of invading viruses by TRIM21 is known as antibody-dependent intracellular neutralization (ADIN), a rapid and efficient process [11]. It is important to highlight that among FcRs, TRIM21 has the highest known affinity for IgG, but also binds to IgA [12] and IgM [13]. TRIM21 is particularly important for the antiviral response, because it mediates the degradation of IgG-coated viruses by proteasomes, and the resulting fragments can activate the antiviral innate immune response [14]. The initial sequence of monoubiquitination is still under debate, as experimental in vitro conditions may profoundly affect the ubiquitination stage. The current model of TRIM21 activation predicts that after dimerization, TRIM21 interacts with the E2 enzymes UBE2W or UBE2D2, which are more promiscuous E2 enzymes [15], promoting the monoubiquitination of TRIM21 at its N-terminus. Then, UBE2N and UBE2V2 build a polyubiquitin chain on the N-terminal monoubiquitin. This step is thought to trigger immune signaling and the proteasomal degradation of TRIM21, IgG, and the virus [16]. Then, the ATPase p97/valosin-containing protein (VCP), an enzyme with segregase and unfoldase activity, is required for TRIM21-dependent proteasome degradation of viral capsids [17]. Finally, after deubiquitination by Poh1 [14,18], proteasomal activity generates cytoplasmic fragments of virus components, including genomic sequences of either DNA or RNA, depending on the pathogen. These immunological danger signals are then recognized by cytosolic pattern recognition receptors (PRRs), such as cGAS, RIG-1, and MDA5. Activating these and other danger sensors leads to an antiviral response and the secretion of inflammatory cytokines, chemokines, and other mediators that ultimately contribute to infection control [19]. In the context of COVID-19, the literature has demonstrated the role of TRIM21 in promoting the ubiquitination of the SARS-CoV-2 nucleocapsid. Then, polyubiquitination results in the tagging of the N protein for degradation by the host cell proteasome. The alpha, beta, gamma, delta, and micron variants can be subjected to TRIM21-dependent degradation by the ubiquitin–proteasome system (UPS) [20].

In this work, we used publicly available scRNA-seq data for an exploratory study on the repertoire of intracellular FcRs in adaptive and innate lymphoid cells (ILCs) besides myeloid cells in bronchoalveolar lavage fluid (BALF). The samples were obtained from healthy individuals and COVID-19 patients with mild or severe symptoms. When comparing the groups of sick patients for *FCGRT* transcription, there was no statistical significance between patient groups when comparing any cell population. On the other hand, *TRIM21* transcription was upregulated in myeloid cells from mild cases compared to the other cell populations and groups. Therefore, we investigated the TRIM21 signaling pathway in the mild group, as it could serve as a protective mediator. Myeloid and adaptive lymphoid cells transcribed the components of the ubiquitin–proteasome system (UPS) and, although the PRRs RIG-1 (*DDX58*) and MDA5 (*IFIH1*) were transcribed, *NLRP3* and some critical components of the NF-κB pathway were not observed. Accordingly, most cells in mild cases were negative or had low *IL1B*, *IL18*, and *TNF* transcriptional levels. These results suggest that, although in a limited number of cells, the TRIM21 FcR may protect COVID-19 patients on two fronts: conventional virus destruction by proteasomes in adaptive lymphoid and myeloid cells and downregulation of the pathway’s inflammatory branch, preventing an exacerbated inflammatory response.

## 2. Results

### 2.1. Identification of Cell Populations

**Figure 1:**
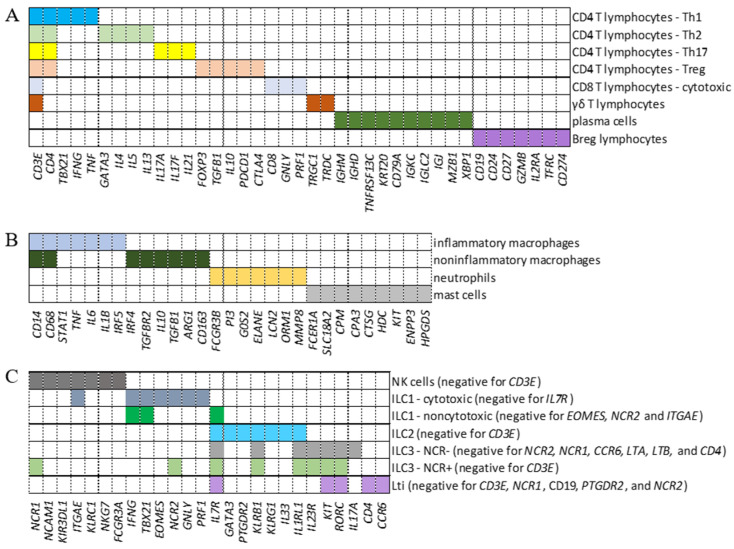
Identification of cell populations. Panels (**A**–**C**) show the combinations of genes used to identify adaptive lymphoid cells (**A**), myeloid cells (**B**), and innate lymphoid cells (ILCs) (**C**), respectively. Each colored segment shows the combination of genes that must be simultaneously transcribed to identify all indicated subpopulations. In the case of ILCs, the genes indicated in parentheses as negative represent a group of additional genes that must not be transcribed for this cellular classification.

To analyze the transcription of intracellular FcRs in adaptive lymphoid cells, myeloid cells, and ILCs from BALF samples, we defined genes whose combination comprised a signature phenotype to identify CD4^+^ Th1, Th2, Treg, Th17, CD8^+^ cytotoxic cells, γδ T lymphocytes, plasma cells, Breg, inflammatory and noninflammatory macrophages, neutrophils, and mast cells (Figure 1A,B). Moreover, ILC1, noncytotoxic ILC1, ILC2, ILC3 NCR, LTi, NK, and ILC3 NCR^+^ cells were identified (Figure 1C). As an exploratory analysis, we wanted multiple cell types included with maximum confidence for cell identification. Considering the numerous genes that could be used to discern each cell population, we first tested two previously published combinations of genes [21,22]. However, when comparing both combinations, the analysis showed an enormous difference in the number of cells identified per population. More significantly, there was a considerable difference in the repertoire of molecules transcribed, such as cytokines and chemokines, besides a lack of transcription of the expected cell markers per population. Identifying myeloid cells was particularly challenging. Then, we decided to design new combinations of marker genes (Figure 1) to identify each cell subpopulation, and used the well-known profile of membrane FcRs for data curation [23], always in control individuals. Then, after numerous tests to ensure that the identified cell subpopulation would transcribe the signature membrane FcRs, the same combinations of genes were used to identify each cell population in COVID-19 patients with mild or severe symptoms.

**Figure 2:**
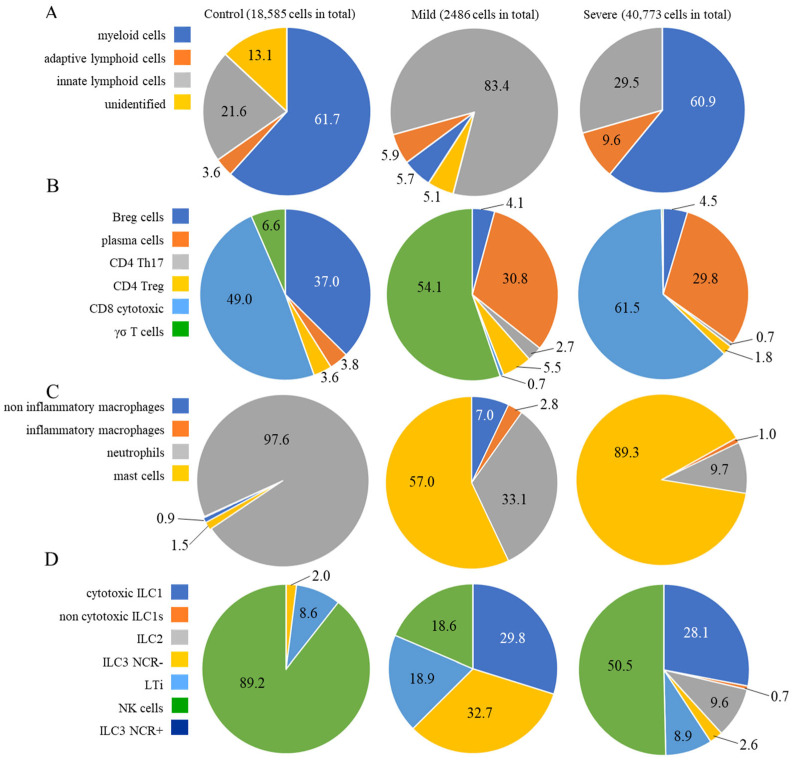
Percentages of different cellular populations among patient groups. The percentage of each cell cluster is shown in (**A**), and the percentages of each subpopulation per cluster are shown in the other panels. Adaptive lymphoid cells are shown in (**B**); myeloid cells are shown in (**C**); and ILCs are shown in (**D**). The total number of cells analyzed per group of patients is shown at the top of the figure.

Approximately 62% of the cells from control individuals were identified as myeloid cells (Figure 2A), with approximately 21% being ILCs and less than 4% being adaptive lymphoid cells (Figure 2A), which were represented mainly by CD8^+^ cytotoxic T and Breg lymphocytes (Figure 2B). Regarding the prevalent population of myeloid cells, neutrophils comprised almost the entire population (Figure 2C), while NK cells were the main ILC population (Figure 2D). In mild cases, approximately 83% of the cells were identified as ILCs, with less than 6% being adaptive lymphoid or myeloid cells (Figure 2A). The main subpopulations were γσ T lymphocytes and plasma cells (Figure 2B), mast cells and neutrophils (Figure 2C), and a similar distribution of ILCs as ILC3 NCR^−^, cytotoxic ILC1, LTi, and NK cells (Figure 2D). In severe cases, almost 61% of the cells were myeloid cells, followed by approximately 30% ILCs and approximately 10% adaptive T lymphocytes (Figure 2A). There were CD8 cytotoxic and plasma cells (Figure 2B), and almost 90% of the myeloid cells were identified as mast cells, with approximately 10% being neutrophils (Figure 2C). Moreover, mostly NK cells and cytotoxic ILC1s were among the ILCs (Figure 2D). The predominant cellular populations found in severe cases were primarily cells with cytotoxic or inflammatory functions, which are correlated with disease severity.

To our knowledge, there is no previous description of ILCs in the lungs of COVID-19 patients. ILCs are crucial for immune defense against infectious pathogens and play an important role in regulating adaptive immunity, tissue remodeling, and repair. They do not rearrange antigen-specific receptors, and although they are found in all tissues, ILCs are particularly enriched in the lungs [24] and mucosal surfaces. In patients with COVID-19, the number of ILCs in peripheral blood decreases greatly in patients with more severe symptoms [25], and clinical recovery is correlated with increased numbers of ILCs, especially ILC2s [26,27,28]. In this work, ILCs were identified based on the literature [29,30].

### 2.2. FCGRT and TRIM21 Transcription

**Figure 3:**
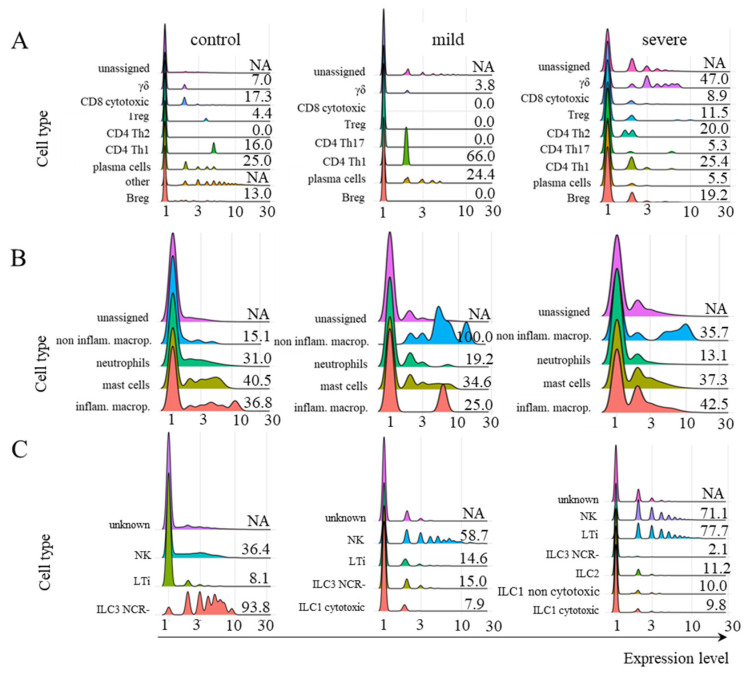
Transcriptional profile of *FCGRT* in different cellular populations. The transcriptional profile of *FCGRT* in the indicated subpopulations of adaptive lymphoid cells (**A**), myeloid cells (**B**), and ILCs (**C**) is shown for control individuals or patients with mild or severe symptoms of COVID-19. The information next to each population represents the percentage of cells whose *FCGRT* transcriptional level was above zero. The colors assigned to each histogram may vary between cell populations, and correct identification should be based on the indicated population name. No CD4^+^ Th2 lymphocytes were identified in the samples from mild cases. To calculate the percentage of FCGRT^+^ cells, we used the number of total cells per subpopulation per patient group (considered 100%). This number was revealed by the phenotypic analysis indicated in the MM section. Then, we determined the number of FCGRT^+^ cells per subpopulation. NA means not available. The statistical analysis returned no significant differences between any cell subpopulation and patient group (considering *p* ≤ 0.05).

After identifying the cell types, we analyzed *FCGRT* transcription in adaptive lymphoid, myeloid cells, and ILC (Figure 3). We observed modest and variable transcriptional levels of this receptor in most cells from all three groups of patients. However, Kruskal–Wallis statistical analysis did not indicate any condition with an expression level statistically higher than the other two conditions simultaneously. The difference between the mean rank of any comparative condition is not large enough to be statistically significant. This conclusion is true of all three cell groups (adaptive lymphoid, myeloid, and ILC). Therefore, we cannot reject the null hypothesis for any cell group in Figure 3 for a significance level of 0.05.

**Figure 4:**
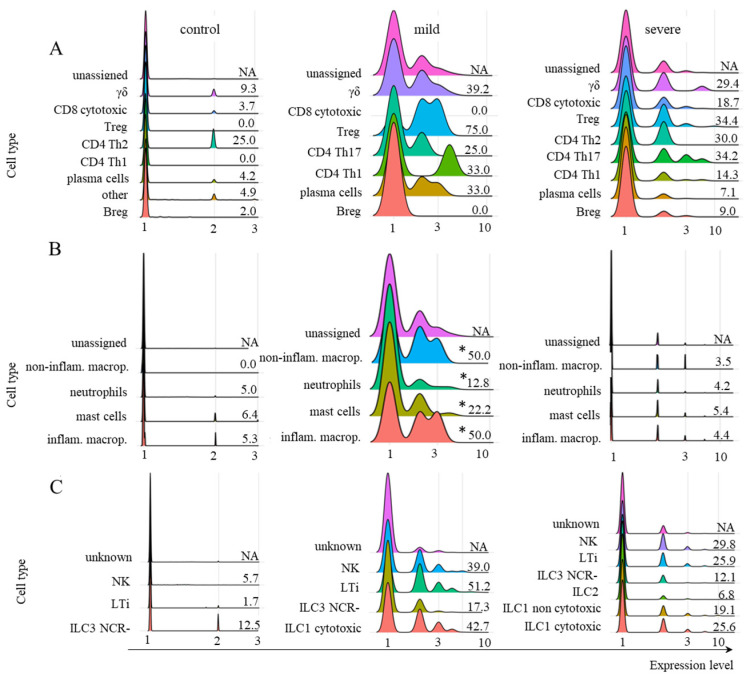
Transcriptional profile of *TRIM21* in different cell populations. The transcriptional profile of *TRIM21* is shown for adaptive lymphoid cells (**A**), myeloid cells (**B**), and ILCs (**C**). The samples were obtained from healthy control individuals or patients with mild or severe symptoms of COVID-19. The information next to each subpopulation represents the percentage of cells with *TRIM21* transcriptional levels above zero. To calculate the percentages, we used the number of total cells per subpopulation (100%) per patient group, revealed by the phenotypic analysis in the MM section, and the number of TRIM21^+^ cells per subpopulation was used. No CD4^+^ Th2 lymphocytes were identified in the samples from mild cases. NA means not available. According to the statistical analysis, only the group of myeloid cells in mild cases was statistically significant when comparing all conditions (* means *p ≤* 0.05).

When we analyzed *TRIM21* transcription, very few adaptive lymphoid cells, myeloid cells, or ILCs from control individuals transcribed this receptor (Figure 4A–C). However, this scenario changed in cells obtained from individuals with mild symptoms, where only Breg and CD8^+^ cytotoxic T cells remained negative for the receptor after infection (Figure 4A–C). In severe cases, the transcription of *TRIM21* reduced in most adaptive lymphoid populations compared to mild cases (Figure 4A) and decreased to near zero in myeloid cells and ILCs (Figure 4B,C). For the myeloid cell group, the Kruskal–Wallis test indicated that the difference between the mean ranks of the mild condition’s expression levels compared to the other two conditions was large enough to be statistically significant. The resulting *p*-value was 0.02277, and the test statistic H equals 7.5649, which is not in the 95% confidence interval ([0, 5.9915]). We found a mean rank score of 5 for control, 10.5 for mild, and 4 for severe cases. The observed effect size η2 is large, 0.62. Therefore, we rejected the null hypothesis for the myeloid cell group (Figure 4).

According to the Kruskal–Wallis statistical analysis, despite the difference in expression levels between the control, mild, and severe conditions, there was no statistically significant difference among the three conditions for adaptive lymphoid and ILC cells for a significance level of 0.05. Therefore, we cannot reject the null hypothesis for these cell groups. Considering adaptive lymphoid cells, although there was no statistical difference between patients with mild or severe symptoms, we analyzed them further, as most cells were transcribing *TRIM21*.

Moreover, the poor transcription of *TRIM21* in myeloid, ILC, and adaptive lymphoid cells from severe cases was confirmed using another dataset [22] (Supplemental Figure S1). The profile and levels of *FCGRT* transcription were also similar in severe cases when comparing both datasets (Figure 3 and Supplemental Figure S1). Considering that *TRIM21* transcription was primarily upregulated in cells from individuals with mild symptoms and that this pathway may be protective against infection, we further investigated its downstream components only in this group of patients. 

### 2.3. Transcription of TRIM21 Signaling Pathway Components

**Figure 5:**
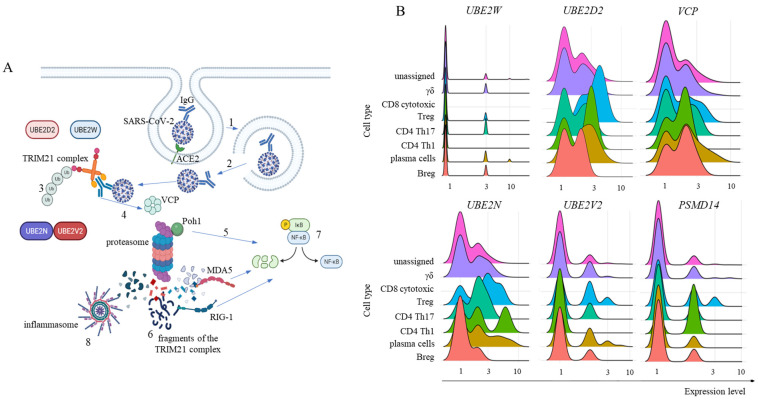
General TRIM21 pathway and its components’ transcription in adaptive lymphoid cells. Panel (**A**) represents the general TRIM21 pathway, where IgG-coated SARS-CoV-2 viruses invade host cells primarily by interacting with the ACE2 receptor and are enclosed in endosomal vesicles (1). Then, the virus bound to IgG must escape the endosome and reach the cytoplasm (2), where TRIM21 dimers bind to the Fc portion of IgG with high affinity. The addition of monoubiquitin is performed via UBE2W or UBE2D2, the structural base for polyubiquitination via UBE2N and UBE2V2 (3). For TRIM21-dependent virus proteasomal degradation, VCP activity is required (4), in addition to substrate deubiquitination by Poh1, which can directly activate the innate antiviral response by activating NF-κB (5). After degradation, cytoplasmic virus fragments (6), including genomic dsRNA recognized by MDA-5 and RIG-1, can also activate NF-κB (7). Moreover, nongenomic fragments of the virus can activate inflammasomes such as NLRP3 (8), all of which contribute to the production of type I interferons and inflammatory cytokines. Panel (**B**) shows our analysis of E2 ubiquitin enzymes *UBE2W*, *UBE2D2*, *UBE2N*, and *UBE2V2* transcriptional levels. Moreover, Poh1 (*PSMD14* gene) and *VCP* transcription were analyzed in adaptive lymphoid cells from COVID-19 patients with mild symptoms. Panel (**B**) shows the transcriptional profile of the indicated molecules in adaptive lymphoid cells from mild COVID-19 cases. The artwork was prepared using BioRender.com.

To activate the TRIM21 pathway, the SARS-CoV-2 virus must be enveloped in IgG and evade endosomal vesicles to enter the cytoplasm (Figure 5A; a general schematic representation of the pathway). It is currently accepted that the binding of TRIM21 to Igs leads to clustering-induced RING domain dimerization. TRIM21 then interacts with the E2 enzyme UBE2W, which first promotes the monoubiquitination of the N-terminus of TRIM21 for UBE2N and UBE2V2 to build a K63-linked polyubiquitin chain. However, it was recently described that UBE2D2 can also initiate polyubiquitination followed by UBE2N and UBE2V2 activity [16] (Figure 5A). Then, some proteasomal substrates require previous enzymatic processing, as in the case of large intact viral capsids. For this purpose, in the TRIM21-dependent virus neutralization pathway, the TPase p97/valosin-containing protein (VCP) is necessary [17]. Furthermore, before proteasomal degradation, ubiquitin(s) must be removed from the substrate, and the proteasome-associated deubiquitinase Poh1 (*PSMD14*) is known to be required for virus degradation [14] (Figure 5A). In fact, Poh1 has dual functions: deubiquitination and induction of the inflammatory response through the activation of NF-κB. Ultimately, multiple viral fragments are generated in the cytoplasm and are made available for danger sensor recognition, such as MDA-5, RIG-1, and inflammasomes, like NLRP3, and others, to trigger the transcription and signaling of multiple inflammatory mediators (Figure 5A).

In ILCs, multiple required components of the pathway were not transcribed by any subpopulation, including *UBE2W*, *UBE2D2*, *UBE2V2*, and *PSMD14*. Therefore, it is unlikely that the TRIM21 pathway plays a protective role exerted by ILCs in mild cases of COVID-19.

Our findings revealed that adaptive lymphoid cells from mild COVID-19 patients had low *UBE2W* transcription levels (Figure 5B). However, most cells from all populations were positive for *UBE2D2* and *UBE2N* transcription, with a moderate general percentage of *UBE2V2*-positive cells (Figure 5B). We also observed that most adaptive lymphoid cells from mild COVID-19 patients transcribed *VCP* (Figure 5B). Regarding Poh1, most Treg, CD4^+^ Th17 cells, and Th1 cells transcribed the *PSMD14* gene. In comparison, fewer γδ cells, plasma cells, and Breg cells were positive for this molecule (Figure 5B).

**Figure 6:**
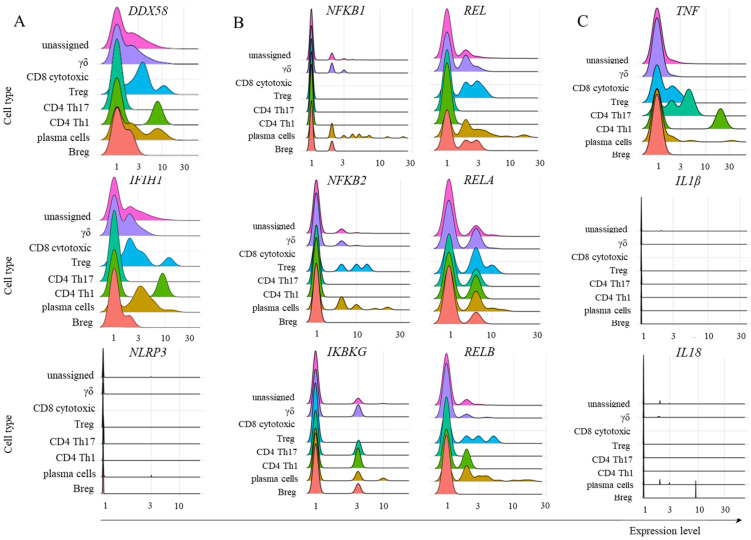
Transcription of some members of the TRIM21-dependent virus innate response. The analysis was performed for the indicated molecules in the populations of adaptive lymphoid cells obtained from mild cases of COVID-19. Panel (**A**) represents the transcription of danger sensors, panel (**B**) represents some members of the NF-κB pathway, and panel (**C**) shows the inflammatory cytokines *TNF*, *IL1B*, and *IL18*.

To evaluate the cognate antiviral response mediated by TRIM21, we studied the transcription of some relevant components involved in sensing immunological danger in the cytoplasm. The PRRs RIG-1 (*DDX58*) and MDA-5 (*IFIH1*) bind to short and long sequences of double-stranded RNA (dsRNA), respectively. SARS-CoV-2 is a retrovirus, so these sensors are expected to play a central role in this response. Moreover, multiple nongenomic viral structures generated by proteasomal degradation are potentially recognized by inflammasomes, and the NLRP3 structure is a pivotal component of this family. In the canonical pathway, inflammasomes can bind to diverse damage-associated molecular pattern (DAMP) or pathogen-associated molecular pattern (PAMP) molecules and, through the activation of caspase 1, activate the inflammatory mediators IL-1β and IL-18 [31]. In the samples obtained from patients with mild symptoms, only CD4^+^ Th17 cells did not transcribe *DDX58* or *IFIH1* (Figure 6A). To our surprise, no adaptive lymphoid population transcribed *NLRP3* (Figure 6A). Considering the NF-κB pathway, we observed no transcription, or a few cells transcribed p105 (*NFKB1*), p100 (*NFKB2*), and *IKBKG* (Figure 6B), whose gene products lead to NF-κB function. The percentage of cells transcribing c-REL (*REL*), p65 (*RELA*), and *RELB* was variable, but generally greater than the percentage of cells transcribing the other components of the NF-κB pathway studied (Figure 6B). Then, the signature cytokines expected after NF-κB and inflammasome activation are TNF and IL-1β/IL-18, respectively. We observed a few Treg cells as TNF^+^ (Figure 6C). Although these cells are associated with IL-10 and TGF-β production, they can secrete TNF under pathogenic conditions [32]. Moreover, no adaptive lymphoid cells transcribed either *IL1B* or *IL18*, suggesting that even if the *NLRP3* gene or any other member of the inflammasome sensor family was being transcribed, the expected outcome of inflammasome activation would likely not be achieved (Figure 6C).

**Figure 7:**
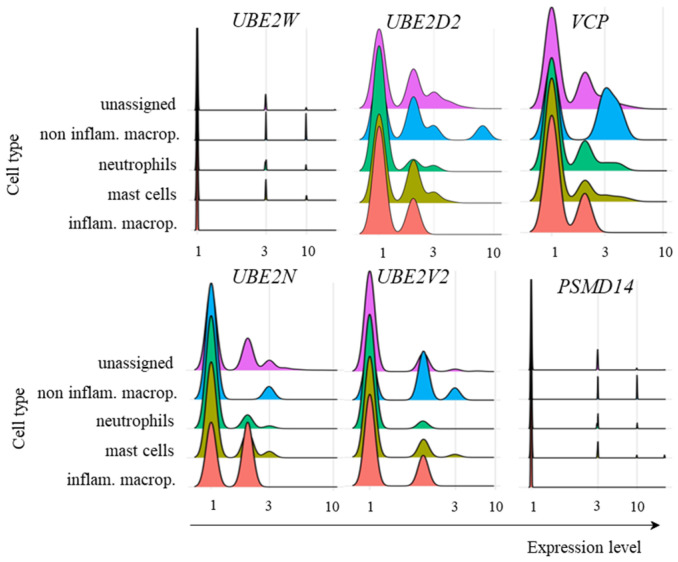
Transcriptional levels of UPS-related genes. The transcriptional levels of the E2 ubiquitin enzymes *UBE2W*, *UBE2D2*, *UBE2N*, and *UBE2V2*, in addition to Poh1 (*PSMD14* gene) and *VCP*, were analyzed in the indicated myeloid cells from COVID-19 patients with mild symptoms.

With regards to myeloid cells from patients with mild symptoms, the transcriptional profile of ubiquitin pathway members was similar to that of adaptive lymphoid cells, with no relevant levels of *UBE2W* transcription and variable levels of *UBE2D2*, *UBE2N*, and *UBE2V2* (Figure 7). *VCP* was also transcribed at different levels by the cell subpopulations (Figure 7). However, *PSMD14* showed no relevant transcription of any population of the myeloid cells analyzed (Figure 7). Presently, it remains unclear whether the absence of Poh1 might obstruct subsequent stages of the pathway, or whether alternative deubiquitinases would act to remove conjugated ubiquitin molecules.

**Figure 8:**
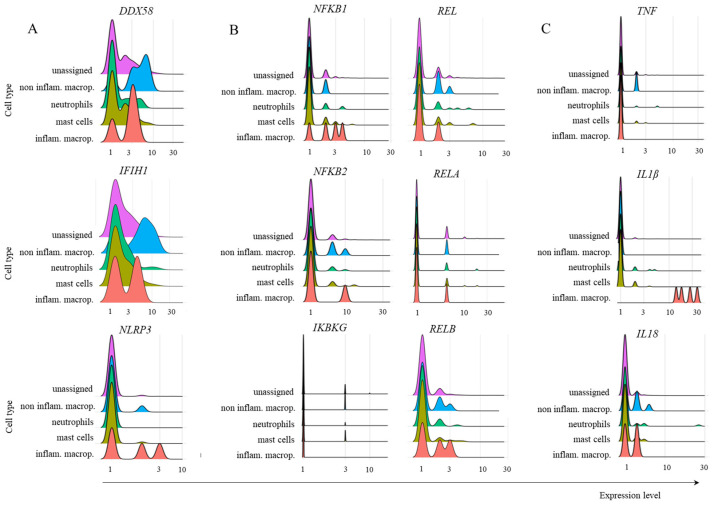
Transcription of several members of the TRIM21-dependent virus innate response in myeloid cells. The analysis was performed for the indicated molecules in the populations of myeloid cells obtained from mild cases of COVID-19. Panel (**A**) represents the transcription of danger sensors, (**B**) represents some members of the NF-κB pathway, and panel (**C**) shows the inflammatory cytokines TNF, IL1B, and IL18.

Multiple myeloid cell populations transcribed *DDX58* and *IFIH1*. However, the *NLRP3* gene was transcribed only by inflammatory macrophages and, at a lower frequency, by noninflammatory macrophages (Figure 8). Most inflammatory macrophages transcribed *NFKB1*, and a few subpopulations transcribed *NFKB2*, *REL,* or *RELA*. Moreover, there was no important transcription of *IKBKG* in any of the cell populations evaluated, and a fraction of cells transcribed *RELB* (Figure 8). Regarding the cytokines, very few noninflammatory macrophages transcribed *TNF*, all inflammatory macrophages transcribed *IL1B*, and most inflammatory and noninflammatory macrophages transcribed *IL18* (Figure 8).

### 2.4. Inflammatory Cytokine Transcription

**Figure 9:**
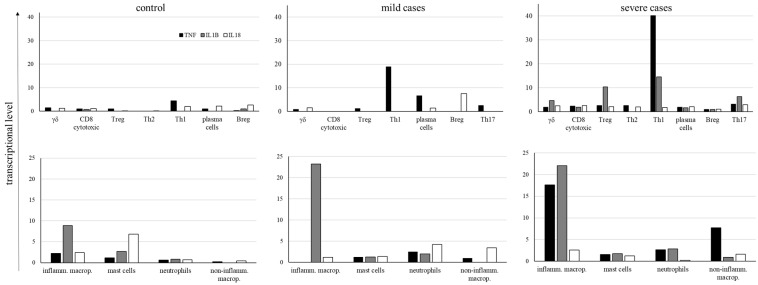
Average levels of cytokine transcription. The levels of *TNF*, *IL1B*, and *IL18* cytokines were analyzed in the indicated cell populations obtained from control individuals and COVID-19 patients with mild or severe symptoms. Negative cells were excluded from calculating cytokine average transcriptional levels, and only cells transcribing each cytokine were considered for the calculation.

Considering the great importance of TNF, IL-1β, and IL18 in the pathogenesis of COVID-19, and in order to evaluate whether their levels were lower in mild cases than in severe cases, we analyzed their average expression levels in all groups of patients (Figure 9). In the control group, very low levels of cytokine transcription were observed in adaptive lymphocytes as well as in myeloid cells, although inflammatory macrophages and mast cells had some prominence (Figure 9). In mild cases, most CD4^+^ Th1 and Th17 lymphocytes transcribed *TNF*, with Breg cells transcribing *IL18*. Moreover, inflammatory macrophages primarily transcribed *IL1B*. As this cytokine must be enzymatically cleaved, we do not know the levels of biologically active IL-1β. On the other hand, in severe cases, comparatively higher levels of *TNF* were found in CD4^+^ Th1 cells in addition to inflammatory and noninflammatory macrophages (Figure 9), with higher levels of *IL1B* in inflammatory macrophages (Figure 9). The extended profile of cytokines and chemokines transcribed by adaptive lymphoid and myeloid cells is shown in Supplemental Figure S2, confirming the increased transcription of inflammatory mediators in severe cases.

## 3. Discussion

A more comprehensive and detailed analysis of FcRs can yield benefits not only for the investigation of SARS-CoV-2 infection, but also of multiple diseases whose treatments involve antibody-based immunotherapies, such as cancer [33], Ebola, cytomegalovirus, influenza, human immunodeficiency, respiratory syncytial viruses [34], psoriasis [35], and many others. Our results indicate that myeloid cell populations found in the lungs of COVID-19 patients with mild symptoms upregulate the TRIM21 receptor and transcribe its required downstream signaling components dedicated to virus degradation. On the other hand, in severe cases, fewer cell populations transcribed the receptor, and its downstream components had mostly near-zero transcriptional levels. Therefore, we hypothesize here that this FcR may contribute to protection through virus degradation in UPS, while leading to no exuberant inflammatory reactions. However, the biological relevance of TRIM21 and FcRn remains to be elucidated in biological assays.

Early in the pandemic, risk factors such as diabetes, hypertension, obesity, cardiovascular diseases, asthma, kidney disease, and chronic obstructive pulmonary disorder were recognized as contributing factors to disease severity. However, it later became clear that an excessive and exuberant inflammatory response could contribute more decisively to increased morbidity and mortality rates [36]. These events ultimately lead to exudative diffuse alveolar damage and end-stage acute respiratory failure. Therefore, understanding the molecular pathways that may contribute to immunoprotection is critical to understanding the pathology.

To illustrate the complexity of the possible roles of FcRs, antibody-dependent enhancement (ADE) is a mechanism by which the severity of certain viral infections is increased in the presence of subneutralizing or nonneutralizing antiviral antibodies. In vitro assays of ADE associate this enhanced pathogenesis with abnormal FcγR-mediated viral host cell invasion, which overlaps with the conventional function of FcγRs in mediating protection [37]. The presence of membrane-bound FcRs in non-COVID-19 patients might be related to pathology, at least in some acute respiratory syndrome coronavirus (SARS-CoV) patients [38]. In this case, critically ill patients who eventually died of SARS had accumulated pulmonary proinflammatory mediators, no wound-healing macrophages, and faster neutralizing antibody responses. Serum from these patients enhanced in vitro SARS-CoV-induced CCL2 chemokine and IL-8 production via human monocyte-derived wound-healing macrophages. In this case, this effect was reduced after a FcγR blockade [39]. Therefore, screening for the biological functions of FcRs and their potential roles in the protection or pathogenesis of infections is central.

Our findings were derived from the analysis of scRNA-seq data, an important method for screening cellular transcriptional profiles. This method enables the assessment of phenotypic modulations and potential biochemical pathways that may play a role in various biological processes. However, confirming the biological relevance of these hypotheses in vitro and in vivo is necessary. Our analysis focused on patients with mild symptoms. In this group, we found a significant transcriptional upregulation of *TRIM21* and a complete repertoire of downstream components for virus destruction. However, our analysis included a limited sample size of only three patients with mild symptoms, accounting for 2486 cells. Multiple reasons led to this reduced sampling. The lung cells were obtained by BAL, an invasive procedure usually not clinically indicated for patients with no severe symptoms. Moreover, multiple parameters must be evaluated and considered when analyzing the different datasets available in public repositories. These include the clinical characteristics used for patient categorization with mild or severe symptoms, clinical procedures to collect the samples, post collection computational processing, data compatibility to run by CellHeap, available datasets containing BALF samples from the three groups of patients, and others. These facts were determinants in analyzing just this dataset of patients with mild disease. As more datasets are available with samples from only severe patients, we analyzed another dataset containing only this group (Supplemental Figure S1). This additional dataset comprised approximately 40,000 cells from twenty patients, and we observed low intracellular FcR profiles, similar to our results for severe patients.

The Kruskal–Wallis test [40] is a nonparametric method, i.e., it does not assume a normal distribution of the residuals, unlike the analogous one-way analysis of variance (ANOVA). If the data contains potential outliers, if the population distributions have heavy tails, or if they are significantly skewed, as frequently occurs with noisy transcriptomic data [41], the Kruskal–Wallis test is more powerful at detecting differences among groups than other tests, such as the ANOVA F-test [42].

Kruskal–Wallis transcriptomic analysis, i.e., the Kruskal–Wallis test in RNA-seq or scRNA-seq data analysis, is used in the literature mainly to compare gene expression levels among groups [43,44,45]. For the statistical analysis of transcriptomic data, we can use the percentage of gene expression to describe the number of genes detected or the frequency of single cells that transcribe a given gene (as in this work) for several transcriptome quality metrics [46,47,48].

The number of immune cells in the lungs of normal individuals was calculated to be in the range of 7 × 10^10^ cells [49]. We do not know the number of total cells in the lungs of patients with mild COVID-19, but this number is possibly greater than that in healthy individuals. With regards to the BAL procedure for cell collection, it probably harvests cells with weaker interstitial adherence, and in our analysis, we excluded virus-infected and dead cells, likely altering the proportion of cell populations found in the lungs. Therefore, reaching an approximate number of lung cells from mild COVID-19 patients who could express TRIM21 and the required components of the pathway would be speculative. However, as an exercise, we observed that 32% of all cells analyzed in mild cases from the three patients transcribed the *TRIM21* gene, and that part of these cells had the downstream components. If as few as 10% of the cells were equipped with all receptor signaling components, and considering the total number of cells in normal lungs as a reference, we would find at least 2.3 × 10^9^ cells per individual who could respond to the infection through TRIM21.

Regarding cellular identification, there is no consensus on the repertoire of genes sufficient to discern individual subpopulations. For mast cells, for example, Liao et al. [21] used the *TPSB2* gene, while Wauters et al. used the genes *MS4A2*, *TPSAB1*, and *TPSB2* [22]. We used both possibilities to identify this population, and the resulting number of cells and transcriptional levels of some selected genes were very different. Moreover, there was no transcription of *FCER1A* in either analysis, a signature gene for mast cells. Therefore, in this work, we used the well-known repertoire of membrane FcRs expressed by each cell population in the group of healthy control patients as an internal reference for data curation. In the case of mast cells, for example, the combination of genes used here identified cells that transcribed *FCER1A*, and the same was carried out for all populations.

The pulmonary landscape of cellular subpopulations of the immune system and their regulated functions are central to the clinical outcome of COVID-19, as populations generally associated with a protective role in viral infections may turn into pathogenic cells. Many aspects related to the abnormal activation of inflammatory pathways in the lungs of patients with severe symptoms remain to be clarified to avoid secondary inflammatory damage and worsened symptoms. For example, in severe cases, a significantly greater percentage of CD4^+^ T lymphocytes producing IFN-γ, TNF, and IL-2 and of CD8^+^ T lymphocytes producing IFN-γ, TNF, and exposed CD107a were observed compared with those in the corresponding mild group [50]. Increasing evidence indicates that Th17 lymphocytes contribute to severe lung pathology and mortality [51], and increased Th2 cytokines are observed in patients with fatal infection [39]. Moreover, virus-specific T lymphocytes obtained from patients with severe COVID-19 tended to have a greater frequency of the central memory phenotype (CD27^+^/CD45RO^+^). On the other hand, the cytolytic activity of NK cells is associated with SARS-CoV-2 clearance and disease control [52]. Plasma cells are also essential for a protective immune response against COVID-19, with strong T lymphocyte responses correlated with increased neutralizing antibodies. Myeloid cells are also pivotal for disease control, and abnormal cells are associated with the death of patients with severe COVID-19. These cells are associated with damage in multiple organs induced by the secretion of high amounts of inflammatory cytokines and chemokines, increased reactive oxygen species (ROS) production, and abnormal coagulation [53]. In general, very high levels of cytokines such as IL-6, TNF, IL-1β, IL-6 [54], and IFN-γ [55] are also critical to the pathophysiology of the disease and the development of severe symptoms. Therefore, we focused on analyzing most of these cell populations.

This work evaluated multiple cell subpopulations based on previously described cells involved in either COVID-19-induced acute respiratory distress syndrome (ARDS) or disease control [21,56,57]. However, as our work focused on potentially protective intracellular FcRs, we aimed to include the regulatory lymphocyte populations of Breg and Treg cells. Breg cells have not been extensively investigated in the context of SARS-CoV-2 infection, but it was previously reported that these cells are important for protecting the lungs from respiratory diseases [58]. Moreover, Treg cells were reported to be a protective cell population in COVID-19 patients, with fewer Treg cells in the blood being associated with poor prognosis and an increased likelihood of long-term lung disease [59].

We found that more than 80% of the lung cells obtained from patients with mild COVID-19 were ILCs, with a balanced frequency of ILC3 NCR-, ILC1 cytotoxic, LTi, and NK cells. This would be compatible with the primarily reduced number of these cells observed in blood from COVID-19 patients, which could suggest the migration of ILCs to primary virus-affected target tissues such as the lungs [27]. However, to the best of our knowledge, the pulmonary enrichment of ILCs has not been previously observed in patients with mild COVID-19. Unlike T and B cells, ILCs are innate lymphocytes that do not rearrange antigen receptors or express cell surface markers associated with other lymphoid or myeloid cell subpopulations. There are five major groups of ILCs, which are NK, ILC1, ILC2, ILC3, and LTi cells, with ILC1s, ILC2s, and ILC3s roughly mirroring CD4^+^ Th1, Th2, and Th17 cells, respectively. NK cells, in turn, reflect the functions of CD8^+^ cytotoxic T cells [60]. Although ILC2s were shown to be protective against pneumonia recovery after COVID-19 [26], this population was not found in our samples from mild cases.

In summary, we observed several molecular features that might contribute to controlling lung infection and disease severity, preventing it from escalating to an uncontrollable immune-mediated pathogenic response. We observed evidence of the relevance of the TRIM21-mediated degradation of SARS-CoV-2 by the UPS, considering the receptor itself and the downstream required components observed in adaptive lymphoid and myeloid cells from mild samples. Moreover, although this pathway can provide PAMP fragments recognized by multiple PRRs involved in the antiviral inflammatory response, this pathway branch does not seem active in these cells. Therefore, other pathways likely act in parallel with TRIM21 to downregulate or impair the transcription of the inflammatory components *NLRP3*, *IL1B*, and *IL18* in most cell types studied, including no transcription in ILC populations. Moreover, essential members of the NF-κB inflammatory pathway, another critical player in the TRIM21 inflammatory pathway, were transcribed at low levels, which is consistent with the low transcription of *TNF* found in mild cases. Taken together, our data suggest the participation of TRIM21 in the refined pathophysiology of COVID-19, possibly having a protective role through the degradation of the virus, but leading to no robust inflammation in the lungs.

## 4. Materials and Methods

According to the authors who uploaded the primary dataset [21], there were three healthy controls, three patients with mild symptoms, and six with severe COVID-19. The patients were categorized as severe if admission to an intensive care unit (ICU) and/or invasive or noninvasive mechanical ventilation was necessary. Moreover, severe cases were diagnosed based on one of the following criteria: respiratory distress with respiratory rate ≥ 30 times min^−1^; fingertip oxygen saturation ≤ 93% at resting state; ratio of partial pressure of arterial oxygen to fraction of inspired oxygen (PaO_2_/FiO_2_)  ≤ 300 mm Hg (1 mm Hg  =  0.133 kPa); and obvious progression of lesions in 24–48 h shown by pulmonary imaging >50%. Patients with mild symptoms had fever during cell collection, respiratory symptoms, and moderate infection with bilateral pneumonia, as evidenced by computed tomography (CT) imaging. However, they required no admission to ICU or mechanical ventilation. The median age of patients with mild COVID-19 symptoms was 36 yo, and that of severely ill individuals was 65 yo. After CT analysis and other laboratory tests, control samples were obtained from three healthy individuals free of tuberculosis, tumors, and other lung diseases. The median age was 24 yo. We used another dataset [22] that consisted of BALF samples from 20 patients with severe COVID-19. All patients were mechanically ventilated or received extracorporeal membrane oxygenation; the median age was 61 years. The scRNA-seq data were deposited in the Gene Expression Omnibus (GEO) under the accession code GSE145926 (https://www.ncbi.nlm.nih.gov/geo/query/acc.cgi?acc=GSE145926 (accessed on 13 October 2024)) [21] and raw sequencing reads [22] were deposited in the European Genome-phenome Archive (EGA) under study no. EGAS00001004717 and with data accession no. EGAD00001006893 (https://ega-archive.org/datasets/EGAD00001006893 (accessed on 13 October 2024)).

We deployed the CellHeap [61] processing workflow for scRNA-seq data analysis in the Santos Dumont (SD) Supercomputer (https://sdumont.lncc.br (accessed on 13 October 2024)), which has an installed processing capacity of 5.1 Petaflop/s. The workflow received as input the BALF scRNA-seq data for the main dataset, PRJNA608742, from the NCBI GEO repository (https://www.ncbi.nlm.nih.gov/Traces/study/?acc=PRJNA608742 (accessed on 13 October 2024)). Liao et al. [21] generated this dataset and considered the samples of twelve patients as input data. Three samples corresponded to healthy individuals (control), three were from patients with mild symptoms, and six had severe COVID-19 symptoms. Each sample had two read files for paired-end runs.

We used the 10× Genomics pipeline CellRanger v.6.1.0 [62] with default parameters for sample demultiplexing. Then, the reads were aligned, and gene expression was quantified using the GRCh38 human genome and a SARS-CoV-2 genome (NC_045512) as a reference. The *count* function of the CellRanger software (V8.0) automatically identified the infected cells, and in this work, all reads associated with SARS-CoV-2 received the “sarscov2” prefix. Then, we executed an R script that created two files, separating infected from noninfected cells. We selected only SARS-CoV-2 noninfected cells for all analyses to avoid possible gene expression subversions that intracellular infection could generate. Therefore, analyzing uninfected cells in the lungs would provide a more accurate understanding of the functions of various cell types.

We used the SoupX [63] package version 1.6.2 to estimate ambient RNA contamination for quality control. Moreover, we used the Seurat [64] v4 package to identify and remove low-quality cells according to the following three criteria commonly used for scRNA-seq quality control processing [65]: Unique Molecular Identifier (UMI) count < 301; Genes expressed < 151 and >3000; and >20% mitochondrial RNA.

Each dataset was normalized and scaled with default parameters for clustering analysis using Seurat v4. After normalization, we executed the following steps:

- The *FindVariableGenes* function detected the variable genes using the “vst” method and a number of features equal to 2000;
- The datasets were integrated with Seurat’s *FindIntegrationAnchors* and *IntegrataData* functions by running a canonical correlation analysis (CCA) on each subset. We then performed dimensionality reduction using the PCA and UMAP algorithms;
- In the final step of the clustering process, we calculated a shared nearest neighbor (SSN) graph between all cells through the *FindClusters* function, with a resolution parameter equal to 1.2. To identify markers for every cluster compared to all remaining cells, we used the *FindAllMarkers* function with default thresholds.

We used the CellAssign (version 0.99.21) [66] and Sctype [67] R packages for cell type annotation. The Sctype was used only for the ILCs, since their annotation required support for negative markers, which CellAssign does not provide. The complete list of markers for CellAssign and Sctype for each cell type is provided in Results Section.

We generated ridge plots for the noncomparative analysis of cell types using Seurat’s *RidgePlot* function. Essentially, the Ridge Plot is a density plot, i.e., a representation of the distribution of gene expression. It uses a kernel density estimate to show the probability density function of a gene expression for a given cell type. Therefore, we analyzed transcriptional modulations using a noncomparative strategy to analyze FcRs and downstream molecules involved in immune regulation, virus danger recognition, and inflammatory response. To calculate the average expression values, we used Seurat’s *AverageExpression* function. Using an R script, we filtered cells for each gene of interest and each cell type for which the expression level was above zero. Then, we applied Seurat’s AverageExpression function to the filtered cell sets.

We analyzed the percentage of each cell cluster (adaptive lymphoid cells, myeloid cells, or ILCs) and their subpopulations in the three patient groups. Considering the control group as an example, we considered the total number of cells in the sample as “A_c_”; in this case, there were 18,585 cells, as indicated in the public repository. Then, considering the total number of adaptive lymphoid cells (B_c_), myeloid cells (C_c_), and ILCs (D_c_), the following formulas were used to determine the percentage of each cell cluster: (100*B_c_)/A_c_; (100*C_c_)/A_c_; and (100*D_c_)/A_c_, respectively. The number of unidentified cells (U_c_) was calculated as A_c_ − (B_c_ + C_c_ + D_c_), and the percentage of U_c_ was calculated as (100*U_c_)/A_c_. The same procedure was performed for both groups of COVID-19 patients.

We applied the Kruskal–Wallis test with a Chi-Square distribution for statistical analysis among the three conditions (control, mild, and severe) to evaluate the expression levels of genes *FCRT* and *TRIM21*. For the analysis, we used the percentage of cells expressing one of the genes for each cell type belonging to a group. In the cases where there were no cells in a given subpopulation, we considered that percentage equal to zero.

We considered a significance level (*p*-value) of 0.05 for the results with a Bonferroni correction method, including outliers. We evaluated the null assumption that when selecting an expression value from the three conditions (control, mild, and severe), any condition would have an equal probability of containing the highest expression value for the cell group considered. In other words, the null hypothesis stated that the difference between the mean ranks of the conditions was not big enough to be statistically significant. We used the Kruskal–Wallis Test Calculator from the Statistics Kingdom portal (https://www.statskingdom.com/Kruskal–Wallis-calculator.html (accessed on 13 October 2024)) to perform the analysis, which was carried out for the data shown in Figure 3 and Figure 4. The other results show the levels of downstream TRIM21 components only in the mild group of patients with COVID-19, and were performed to evaluate whether the components were being transcribed (not a comparative analysis).

## Data Availability

The datasets used for all analyses included in this work are publicly available and were deposited in the Gene Expression Omnibus (GEO) under the accession code GSE157344 (https://www.ncbi.nlm.nih.gov/geo/query/acc.cgi?acc=GSE157344) [21].

## Supplementary Materials

The following supporting information can be downloaded at: https://www.mdpi.com/article/10.3390/ijms26062769/s1.
