# Supplementary material for: Pulmonary Myeloid Cells in Mild Cases of COVID-19 Upregulate the Intracellular Fc Receptor TRIM21 and Transcribe Proteasome-Associated Molecules
Source: Int J Mol Sci. 2025 Mar 19;26(6):2769. doi: 10.3390/ijms26062769 (PMC11943277; doi:10.3390/ijms26062769)
Supplement: Supplementary file 1 [file ijms-26-02769-s001.zip › ijms-3333367-supplementary.pdf]

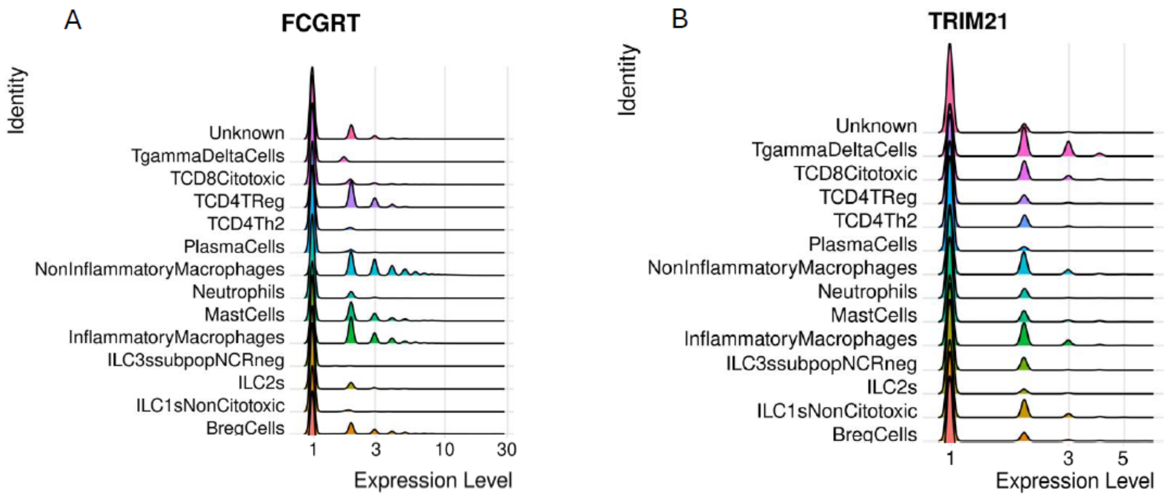

Supplemental Figure S1. Transcriptional profile of FCGRT and TRIM21 in different cell populations. The transcriptional profileof both genesis shown for adaptive lymphoid cells, myeloid cells, and ILCs in a dataset that contains only severe cases. All cell subpopulations identified in this dataset are shown. No more than 9% of each cell population transcribed TRIM21 (B)

CONTROL

	CCL2	CCL3	CCL5	CXCL8	CXCL10	IFNG	IL10	IL17A	IL17F	TGFB
non inflammatory macrophages	+	+	0	+	0	0	+	0	0	+
neutrophils	0	0	0	0	0	0	0	0	0	+
mast cells	+	+	+	+	0	+	0	0	0	++
inflammatory macrophages	0	++	0	++	+	0	0	0	0	++
γδ T lymphocytes	0	+	+++	+	0	+	0	0	0	+
CD8 T cytotoxic	0	+	+++	0	0	+	0	0	0	+
Treg cells	0	+	+	0	0	0	+	0	0	+
CD4 Th2	0	0	0	0	0	0	0	0	0	0
CD4 Th1	0	+	++	++	0	+	0	0	0	+
plasma cells	0	0	+	+	0	0	0	0	0	+
Breg cells	0	0	0	0	0	0	0	0	0	+

0	0
0.1 - 10.0	+
10.1 - 30.0	++
30.1 - 100.0	+++
100.1 - 400.0	++++

MILD

	CCL2	CCL3	CCL5	CXCL8	CXCL10	IFNG	IL10	IL17A	IL17F	TGFB
non inflammatory macrophages	++++	++	+	0	++++	0	+	0	0	+
neutrophils	++	++	0	0	+++	0	0	0	0	+
mast cells	++	+	+	0	++	0	0	0	0	+
inflammatory macrophages	++++	++	0	+	++++	0	0	0	0	+
γδ T lymphocytes	0	0	+++	0	0	0	0	0	0	+
CD8 T cytotoxic	0	0	0	0	0	0	0	0	0	0
Treg cells	0	0	+	0	0	+	+	0	0	+
CD4 Th17	0	0	+	0	0	0	0	+	+	+
CD4 Th1	0	0	+++	0	0	+++	0	+	0	+
plasma cells	0	0	+	0	0	0	0	0	0	+
Breg cells	0	0	0	0	0	0	0	0	0	0

SEVERE

	CCL2	CCL3	CCL5	CXCL8	CXCL10	IFNG	IL10	IL17A	IL17F	TGFB
non inflammatory macrophages	+++	+	++	+	++	++	++	0	0	++
neutrophils	++	++	+	++	++	+	+	0	0	+
mast cells	+++	++	+	+	++	+	+	0	0	+
inflammatory macrophages	++++	+++	++	+++	+++	+	+	0	0	+
γδ T lymphocytes	++++	++	+	+++	+	0	+	0	0	+
CD8 T cytotoxic	++	+	++	+	++	+	+	0	0	+
Treg cells	+++	++	++	+	++	+	++	0	0	+
CD4 Th17	+	+	+	+	+	+	+	0	++	+
CD4 Th2	+	++	+	+	+	0	0	0	0	+
CD4 Th1	+++	++++	+++	+++	+++	++	+	0	0	+
plasma cells	++	+	+	+	+	+	+	0	0	+
Breg cells	+	+	+	+	++	+	+	0	0	+

Supplemental Figure S2. Qualitative analysis of transcription levels. After the identification of each cell population per patient group, the average level of mRNA transcription for each cytokine or chemokine was analyzed. Negative cells were excluded from the calculation and the qualitative results are expressed as indicated in the intensity ranges.
